# The *Arabidopsis* Mitochondrial Nucleoid–Associated Protein WHIRLY2 Is Required for a Proper Response to Salt Stress

**DOI:** 10.1093/pcp/pcae025

**Published:** 2024-03-08

**Authors:** Yuri L Negroni, Irene Doro, Alberto Tamborrino, Irene Luzzi, Stefania Fortunato, Götz Hensel, Solmaz Khosravi, Laura Maretto, Piergiorgio Stevanato, Fiorella Lo Schiavo, Maria Concetta de Pinto, Karin Krupinska, Michela Zottini

**Affiliations:** Department of Biology, University of Padova, Via U. Bassi 58/b, Padova 35131, Italy; Department of Biology, University of Padova, Via U. Bassi 58/b, Padova 35131, Italy; Department of Biology, University of Padova, Via U. Bassi 58/b, Padova 35131, Italy; Department of Biology, University of Padova, Via U. Bassi 58/b, Padova 35131, Italy; Department of Biosciences, Biotechnology and Environment, University of Bari, Campus Universitario, Via Orabona, 4, Bari 70125, Italy; Plant Reproductive Biology, Department of Physiology and Cell Biology, IPK, Corrensstraße 3, Seeland, Gatersleben D-06466, Germany; Plant Reproductive Biology, Department of Physiology and Cell Biology, IPK, Corrensstraße 3, Seeland, Gatersleben D-06466, Germany; Department of Agronomy, Food, Natural Resources, Animal and Environment, University of Padova, Viale Università 16, Legnaro, Padova 35020, Italy; Department of Agronomy, Food, Natural Resources, Animal and Environment, University of Padova, Viale Università 16, Legnaro, Padova 35020, Italy; Department of Biology, University of Padova, Via U. Bassi 58/b, Padova 35131, Italy; Department of Biosciences, Biotechnology and Environment, University of Bari, Campus Universitario, Via Orabona, 4, Bari 70125, Italy; Botanisches Institut, Christian-Albrechts-Universität zu Kiel, Am Botanischen Garten 1-9, Kiel D-24098, Germany; Department of Biology, University of Padova, Via U. Bassi 58/b, Padova 35131, Italy

**Keywords:** *Arabidopsis*, Mitochondria, mtDNA, Nucleoid, Salt stress, WHIRLY

## Abstract

In the last years, plant organelles have emerged as central coordinators of responses to internal and external stimuli, which can induce stress. Mitochondria play a fundamental role as stress sensors being part of a complex communication network between the organelles and the nucleus. Among the different environmental stresses, salt stress poses a significant challenge and requires efficient signaling and protective mechanisms. By using the *why2* T-DNA insertion mutant and a novel knock-out mutant prepared by CRISPR/Cas9–mediated genome editing, this study revealed that WHIRLY2 is crucial for protecting mitochondrial DNA (mtDNA) integrity during salt stress. Loss-of-function mutants show an enhanced sensitivity to salt stress. The disruption of WHIRLY2 causes the impairment of mtDNA repair that results in the accumulation of aberrant recombination products, coinciding with severe alterations in nucleoid integrity and overall mitochondria morphology besides a compromised redox-dependent response and misregulation of antioxidant enzymes. The results of this study revealed that WHIRLY2-mediated structural features in mitochondria (nucleoid compactness and cristae) are important for an effective response to salt stress.

## Introduction

Rapid climate change increasingly exposes plants to novel environmental conditions outside their physiological limits and beyond the range to which they are adapted. In this context, mitochondria, the primary powerhouse of the plant cell and essential hub for many metabolic processes, play an important role. Several metabolic pathways either originate from or converge on mitochondria. Maintaining organelle integrity in terms of functionality, morphology and dynamics is crucial for cellular homeostasis and proper responses to environmental challenges (Liberatore et al. 2016). Properly functioning mitochondria are required for efficient responses to any change in developmental or stress-response processes.

Mitochondria can form dynamic, interconnected networks regulated by an equilibrium of fusion and fission events that, in turn, determine the organelle number, size, shape, dynamics and, most importantly, functionality. The regulation of the mitochondrial network dynamics is critical for energy homeostasis, allowing the plant to respond rapidly and directly to acute metabolic perturbations by balancing energy demand and production (Yu and Pekkurnaz 2018). Alterations of the shape and dynamics of mitochondria are strongly linked to genome instability and can harm the organism (Hoppins 2014, Prevost et al. 2018).

The integrity of mitochondria highly depends on the integrity of their genome, which many factors can compromise. Thus, an active DNA recombination system is crucial, allowing the cell to correct detrimental mutations induced by environmental stress. In particular, high-frequency homologous recombination (HR) and base excision repair systems are required for efficient DNA repair (Marechal and Brisson 2010, Gualberto et al. 2014). Defects in any of these systems can lead to failures in maintaining the stability of the mitochondrial genome, resulting in the accumulation of mutations and in genomic rearrangements that, if not correctly repaired or removed, can become deleterious for the whole organism (Gualberto and Kühn 2014).

One of the most abundant proteins in the mtDNA repair system is WHIRLY2, a member of the WHIRLY DNA-binding protein family that is found in angiosperms (Krupinska et al. 2022). In *Arabidopsis thaliana*, the WHIRLY family is represented by three members: AtWHIRLY1, AtWHIRLY2 and AtWHIRLY3. These proteins are localized in the DNA-containing organelles: WHIRLY1 and WHIRLY3 have been characterized as plastid DNA-binding proteins (Pfalz et al. 2006), while WHIRLY2 is present in mitochondria (Krause et al. 2005). WHIRLY2 is the most abundant single-stranded DNA (ssDNA) binding protein in *Arabidopsis* mitochondria (Fuchs et al. 2020). WHIRLIES could bind ssDNA stretches in a sequence-independent manner (Desveaux et al. 2002). This binding may occur in many parts of the mitochondrial genome, including replication forks (Wyman and Kanaar 2006), promoter regions and, of course, during mtDNA repair processes, where WHIRLY2 binds promoting HR and accurate DNA repair (Cappadocia et al. 2010). WHIRLY2 inhibit end-joining mechanisms by blocking the access of ssDNA 30-OH ends to SSBs and DNA polymerases (Gualberto and Kühn, 2014). By binding to resected DNA ends, WHIRLY2 prevents the annealing of ssDNA overhangs to micro-homologous sequences present in stretches of ssDNA (Marechal and Brisson 2010). This restricts the double-strand break (DSB) repair by error-prone microhomology-mediated break-induced replication, which would generate aberrant mtDNA recombination products negatively impacting plant physiology and reproduction efficiency (Cappadocia et al. 2010). Loss of WHIRLY2 inhibits HR repair of genotoxic-induced breaks, leading to significant rearrangements of the mtDNA, by increased ectopic recombination involving repeats of intermediate size and microhomologies (Janicka et al. 2012, Golin et al. 2020).

Recently, we have demonstrated that WHIRLY2, besides being crucial for mtDNA integrity, is also a nucleoid architectural protein determining nucleoid morphology. The reduced compactness of mitochondrial nucleoids in the *why2-1* mutant was shown to negatively affect the biogenesis of mitochondria as well as their functionality, morphology and dynamics (Golin et al. 2020). Those results show, on the one hand, a link between the mitochondrial nucleoid structure and mitochondria morphology, dynamics and functionality and, on the other hand, that this link exists in plants, as was already shown in human cells (Li et al. 2016).

Recent research showed that the overexpression of *SlWHIRLY2* in tobacco and tomato plants was associated with high resistance to drought stress (Meng et al. 2020) and pathogen attack (Zhao et al. 2018). During drought stress, the overexpression of *SlWHIRLY2* is associated with an enhanced capacity of the reactive oxygen species (ROS) scavenging system and reduced ROS accumulation. The authors suggested that SlWHIRLY2 overexpression does enhance the tolerance to drought stress via transcriptional regulation of mitochondrial genes and stabilization of mitochondrial functionality (Zhao et al. 2018).

The present work shows that in *A. thaliana*, a loss of WHIRLY2 functionality increased the sensitivity of plants to salt stress compared with wild-type (WT) plants. Experimental evidence revealed that WHIRLY2 intervenes in orchestrating the cellular response to salt stress, both by its role in mtDNA repair and by its architectural impact on nucleoids that might function as a recruitment platform for other proteins involved in the maintenance of mitochondria integrity and the regulation of stress signaling pathways.

## Results

### CRISPR/Cas9 mutant line characterization

**Fig. 1:**
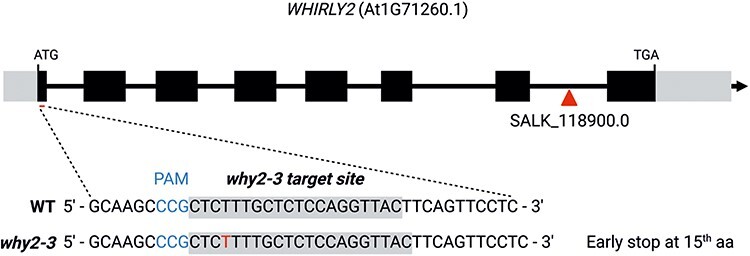
Generation of *why2-3* knock-out mutant with the CRISPR/Cas system. Overview of the WHIRLY2 locus and target sites for the sgRNA-Cas9 complex. Rectangles and lines depict exons and introns in *WHIRLY2* gene, respectively. A spacer sequence was designed *in silico* to target the sgRNA-Cas9 complex to the corresponding targets site (underlined) in Exon 1. Protospacer adjacent motif sequences are reported (PAM). Expected protein is interrupted at the 15^th^ amino acid. T-DNA insertion site of *why2-1* mutant is indicated by a triangle.

To evaluate the role of WHIRLY2 in stress response, two loss-of-function mutants were employed: the T-DNA insertional lines *why2-1* available at ‘The Arabidopsis Information Resource’ (SALKseq_118900.0) and the *why2*-3 mutant lines generated by CRISPR/Cas9 mutagenesis. The *why2-1* line has been already characterized (Cappadocia et al. 2010, Golin et al. 2020), while the *why2-3* line was characterized in this work. In this latter line, a T insertion mutation is introduced, leading to a shift in the reading frame that results in the generation of a stop codon in the first exon upstream of the WHIRLY domain (**Fig. 1**). The characterization of the new mutant line compared with WT and *why2-1* mutant is reported in **Supplementary Fig. S1**. The *why2-3* phenotype resembles the one of *why2-1*. No obvious phenotype was observed during the vegetative growth under standard conditions, as revealed by the primary root length (**Supplementary Fig. S1A**), the primary inflorescence and rosette development (**Supplementary Fig. S1D, E**). Still, a reduction of about 25% of seed germination at 72 h was observed (**Supplementary Fig. S1B**), similar to the *why2-1* (Golin et al. 2020). In the absence of WHIRLY2, mitochondria of *why2-3* appeared elongated (**Supplementary Fig. S1C**). It has been confirmed that the disruption of *WHIRLY2* increased the sensitivity to the genotoxic agents. *Arabidopsis* plants from the three genotypes (WT, *why2-1* and *why2-3*) were grown in a solid medium supplemented with ciprofloxacin at different concentrations, and the amount of mtDNA was analyzed by using opposite direction primers as previously described (Cappadocia et al. 2010). As shown in **Supplementary Fig. S1F**, in both mutant lines, aberrant recombination products accumulated more than in WT, where aberrant products were observed only in the presence of the highest concentration of ciprofloxacin. Based on these results, the two mutant lines were considered equivalent.

### *cis*-Regulatory element analysis

To gain information on the possible role of WHIRLY2 in stress responses, an in silico analysis of the gene’s *cis*-regulatory elements was performed. For the analysis, the 344-bp region upstream of the gene and the first exon and first intron were considered. The inclusion of the first exon and first intron was done since they could also contain important regulatory sequences (Schmitz et al. 2022).

**Fig. 2:**
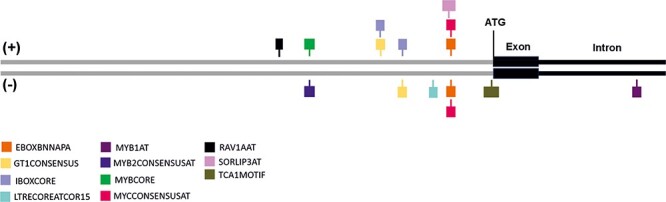
Putative *cis*-regulatory elements of *WHIRLY2*. Position of the different transcription factor binding sites found by the use of the PLACE database. The gray line represents the 344-bp region upstream of the start codon, the black box the first exon and the black line the first intron of the gene. Each colored box represents a different *cis*-element, with the width corresponding to the sequence length and its position on the sense (+) or antisense (+) strand.

The analysis revealed the presence of 11 *cis*-regulatory elements, some of which are associated with abiotic stress responses (**Fig. 2**, **Table 1**). In particular, MYB1AT, MYB2CONSENSUSAT and MYCCONSENSUSAT are binding sites of MYB and MYC transcription factors that participate in the regulation of many different stress responses (Baldoni et al. 2015). They can be found in the promoter of the *RD22*, a dehydration-responsive gene, associated with drought and water stress (Abe et al. 2003). Similarly, MYBCORE is also a binding site for two MYB transcription factors, AtMYB1 and AtMYB2, that have been shown to be involved in dehydration stress response (Urao et al. 1993) as well as AtMYB49 that is associated with an increased salt resistance (Zhang et al. 2020). The RAV1AAT sequence is the binding site of RAV transcription factors and has been found to participate in the regulation of drought and salt stress response and in the response to pathogen attacks (Sohn et al. 2006). The analysis also highlighted the presence of the LTRECOREATCOR15, which is a regulatory element associated with cold and drought response (Baker et al. 1994), and TCA1MOTIF, which is related to biotic and abiotic stresses response and salicylic acid–regulated expression (Li et al. 2020). Finally, several binding sites for light-induced transcription factors (EBOXBNNAPA, GT1CONSENSUS, IBOXCORE and SORLIP3AT) were also found, suggesting a possible involvement of WHIRLY2 in different cellular processes. The presence of these *cis*-regulatory elements in the *WHIRLY2* gene supports the likelihood that WHIRLY2 is involved in abiotic stress responses in *Arabidopsis*. Particularly, the presence of several elements associated with drought, water and salt stress aligns with the findings in tomato by Zhao et al. (2018), hinting at the involvement and potential role of WHIRLY2 in salt stress response in *Arabidopsis*.

**Table 1:** Putative *cis*-regulatory elements present in the 344-bp region upstream of *WHIRLY2*, first exon and first intron

*cis*-Acting element	Sequence	Number of repeats	Localization	Description	Reference
EBOXBNNAPA	CANNTG	2	−26	Light-response element	Hartmann et al. 2005
GT1CONSENSUS	GRWAAW	3	−60; −75; −156	Light-response element	Zhou, 1999
IBOXCORE	GATAA	2	−61; −76	Light-response element	Terzaghi and Cashmore, 1995
LTRECOREATCOR15	CCGAC	1	−38	Temperature and drought stress–response element	Baker et al. 1994
MYB1AT	WAACCA	1	+98	Dehydration-response element	Abe et al. 2003
MYB2CONSENSUSAT	YAACKG	1	−125	Dehydration-response element	Abe et al. 2003
MYBCORE	CNGTTR	1	−125	Dehydration and salt stress–response element	Urao et al. 1993; Zhang et al. 2020
MYCCONSENSUSAT	CANNTG	2	−25; −25	Dehydration-response element	Abe et al. 2003
RAV1AAT	CAACA	1	−147	Drought and salt stress– and abiotic stress–response element	Sohn et al. 2006
SORLIP3AT	CTCAAGTGA	1	−25	Light-response element	Hudson and Quail, 2003
TCA1MOTIF	TCATCTTCTT	1	+1	Biotic and abiotic stress–response element	Li et al. 2020

The list was obtained by analyzing the sequence using the PLACE database. The position of the elements is reported from the translation start site (−upstream, +downstream)

### WHIRLY2 loss of function increased salt stress sensitivity in *Arabidopsis*

**Fig. 3:**
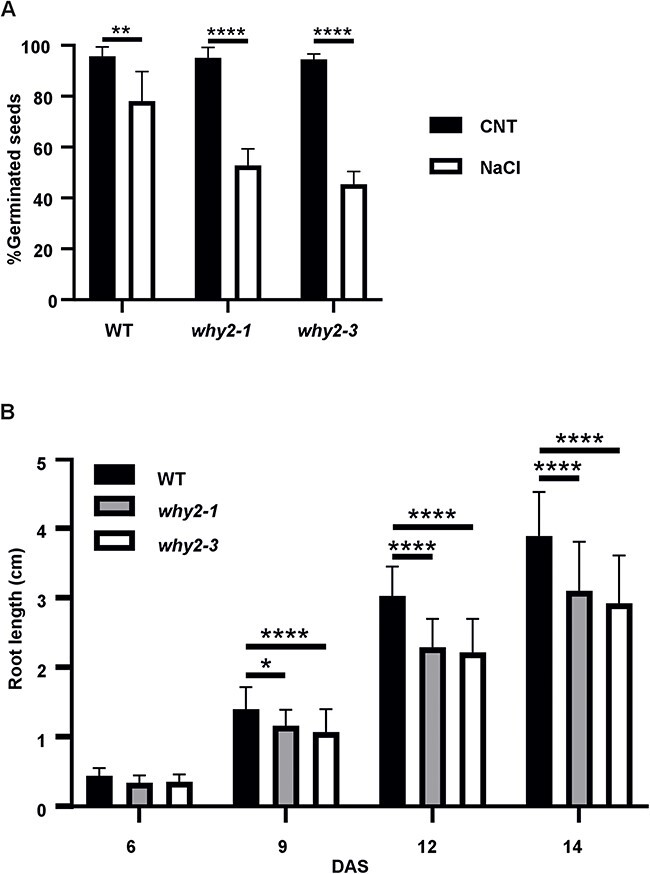
*Why2* mutants are highly sensitive to salt stress. (A) Seed germination percentage and (B) primary root growth of *why2-1, why2-3* and WT sown in solid medium supplemented with 100 mM NaCl. Statistical significance (*P*-value from the *t*-test) compared to non-treated samples [in (A)] or WT [in (B)] is indicated by asterisks (*P < 0.05; **P < 0.005; ***P < 0.001; ****P < 0.0001). Error bars: SE (Standard Error).

To functionally characterize WHIRLY2 and to test its role in stress response, the T-DNA insertion line (*why2-1*) and the *why2-3* mutant were exposed to salt stress to evaluate their sensitivity in terms of germination efficiency and primary root length. It was observed that both parameters were significantly reduced in stress conditions. Still, there was a significant difference between WT and mutants: the reduction in germination efficiency at 96 h was about 44–49% in the two mutants compared to 24% in WT (**Fig. 3A**), and the primary root length was about 18–26% lower in the two mutants than in WT (**Fig. 3B**). Based on these results, the two mutants showed an equivalent response to salt treatment, and thus the following experiments were conducted using the *why2-1* mutant.

**Fig. 4:**
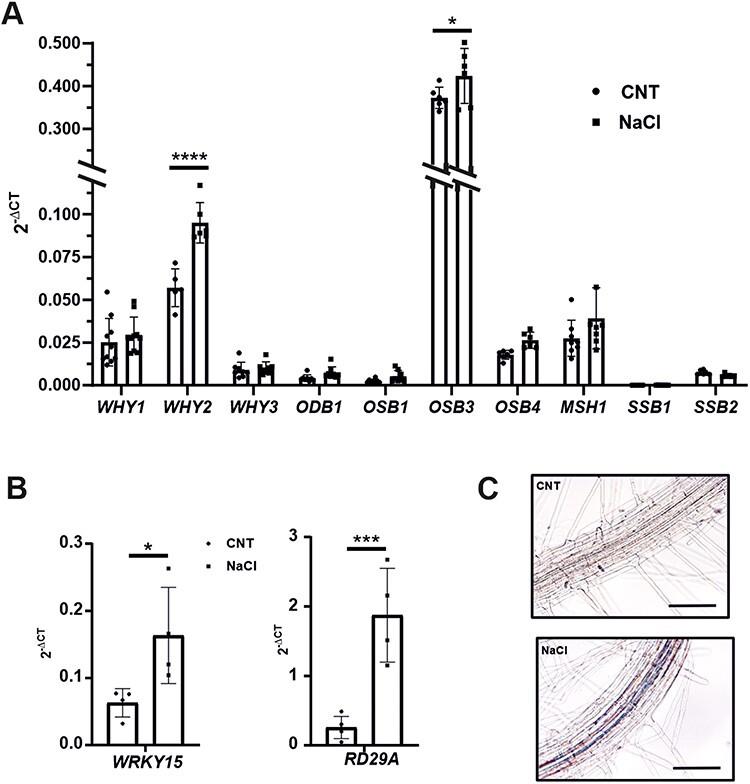
WHIRLY2 is specifically induced by salt stress. Expression of genes coding for mitochondrial ssDNA binding proteins (A) and of stress-marker genes (B) in 14 DAS seedlings treated for 8 h with 150 mM NaCl. *ACT2* has been used as housekeeping gene. The results were analyzed using the 2^(−ΔCt^) method. Error bars: SE. Statistical significance (Tukey’s multiple comparisons test) compared to non-treated samples is indicated by asterisks (*P < 0.05; **P < 0.005; ***P < 0.001; ****P < 0.0001). (C) Expression patterns of the *WHIRLY2 promoter:β-GUS* in root of 7-day-old seedlings. Bar 100 µm.

To evaluate the expression profile of *WHIRLY2* during salt stress, WT plants were grown for 14 d in standard solid medium and subsequently exposed to salt (150 mM NaCl) for 8 h before collecting them for quantitative reverse transcription (qRT)-PCR analysis. The chosen salt concentration and the duration of the treatment were previously shown to substantially inhibit root growth without causing seedling death (Geng et al. 2013). After 8 h of exposure to salt, *WHIRLY2* expression was significantly induced (**Fig. 4A**) and, at the same time, a stress signal was generated. This was evidenced by the upregulation of *WRKY15* (**Fig. 4B**), an early hydrogen peroxide (H_2_O_2_)–responsive transcription factor playing a key role in salt stress response (Vanderauwera et al. 2012), and *RD29A* (**Fig. 4B**), a marker gene for drought/salt stress (Lee et al. 2016).

To determine spatial and stress-associated expression patterns of *pWHIRLY2*, the *WHIRLY2 promoter:β-glucuronidase* (GUS) reporter gene fusion construct was examined. In 14-day-old seedlings treated as described earlier, a solid blue staining was observed in the perivascular tissue of the primary root (**Fig. 4C**), indicating the specific induction of the *WHIRLY2*.

It is worth noting that, within the *WHIRLY* gene family, only *WHIRLY2* was induced by salt stress. At the same time, the expression of other members, *WHIRLY1* and *WHIRLY3*, did not show any significant variation (**Fig. 4A**). In addition, there was no significative induction of genes coding for other proteins involved in mtDNA repair as *ODB1, MSH1, OSB1-4* and *SSB1* and *SSB2*. A slight induction has been observed in the gene coding for the mitochondria/chloroplast-localized OSB3 (**Fig. 4A**).

### WHIRLY2 ensured mtDNA integrity during stress

**Fig. 5:**
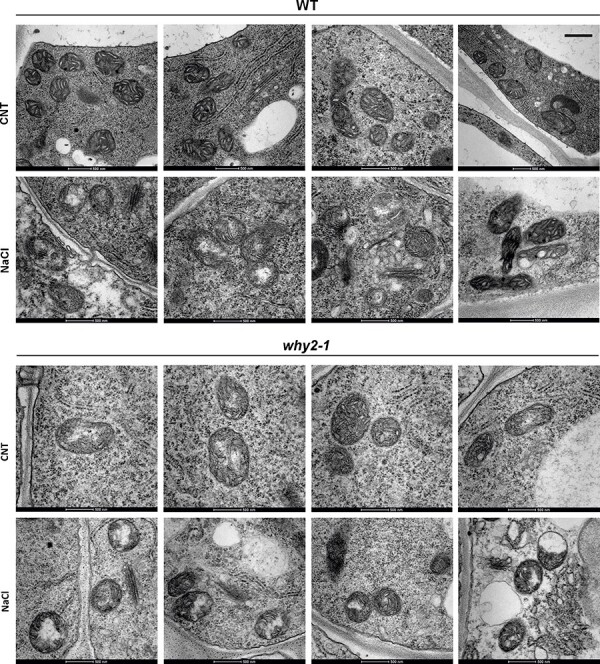
WHIRLY2 disruption affects mitochondrial and nucleoid organization. Transmission electron microscope images of WT and *why2-1* root epidermal cells of seedlings grown for 23 d on solid medium with or without 100 mM NaCl. Bar 500 nm.

**Fig. 6:**
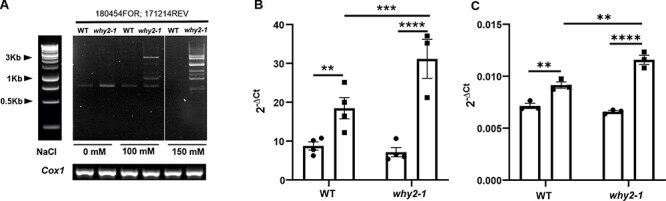
*Why2-1* mutants accumulate aberrant recombination products during salt stress. (A) Electrophoretic analysis of representative PCR performed with two inward-facing mitochondria genome–directed primers. The *Cox1* mitochondrial gene was used as loading controls. The oligonucleotides used for each PCR are indicated. Plants were grown for 23 DAS in normal solid medium under different concentrations of salt (0, 100 or 150 mM NaCl). The experiment was repeated three times, and 36 plants per biological replicate were used. (B) mtDNA copy number calculated on the level of *orf170mito* (mitochondrial RNA–dependent DNA polymerase). *RPOTP* (nuclear plastid-RNA polymerase) has been used as housekeeping gene. (C) Relative expression of *PolIB*. ACT2 has been used as housekeeping gene. The results were analyzed using the 2^(−ΔCt)^ method. Statistical significance (*P*-value from Student’s *t*‐test and Tukey’s multiple comparisons) is indicated by asterisks (*P < 0.05; **P < 0.005; ***P < 0.001; ****P < 0.0001). Error bars: SE.

To evaluate the impact of WHIRLY2 disruption on mitochondrial morphology in salt stress conditions, WT and mutant plants were grown for 23 d on a standard solid medium containing different concentrations of NaCl, and the root mitochondria were analyzed using transmission electron microscopy (TEM). As reported in **Fig. 5**, salt treatment induced alterations of mitochondrial morphology in both WT and mutant plants. In the WT plants, under salt treatment, some mitochondria exhibited fewer cristae than in control conditions along with the presence of a translucent area, associated with nucleoid disassembly (Golin et al. 2020). In mutant plants, it has been confirmed that mitochondria contained fewer cristae than WT already under control conditions (Golin et al. 2020). Upon exposure to salt stress, mitochondria morphology was severely compromised. Due to an almost complete absence of cristae, the organelles appeared swollen and low electron-dense, while the translucent area, indicative of a disorganization of nucleoids, appeared to be enlarged. To evaluate the impact of WHIRLY2 on mtDNA integrity in salt stress conditions, PCR analyses were performed on specific mtDNA regions using opposite direction primers. In WT mitochondrial genome, these primers are expected to yield no product. However, in a rearranged genome with circular or head–tail concatemers, new fragments containing both extremities of the amplified regions would be present (Marechal and Brisson, 2010). As reported in **Fig. 6A**, many amplicons correspond to aberrant rearrangements accumulated in the mitochondria of mutant plants upon salt stress treatment. WT plants showed a low level of aberrant products even after exposure to the highest salt concentration. After 10 d of recovery in standard medium without salt, aberrant recombination products were no more visible in WT but still evident in the mutants (**Supplementary Fig. S2**). It has been observed that salt treatment induced an increase in mtDNA content (**Fig. 6B**) associated with an augmented expression of *PolIB* (**Fig. 6C, D**), likely due to an increase in the energy demand to cope with the stress. Interestingly, in the mutant, this increase was higher than that in WT plants. This result is consistent with an increased mtDNA replication, partially induced by an increased recombination frequency triggered by DSB occurring in mtDNA, as also reported by Parent et al. (2011) who demonstrated that *PolIB* either evolveda specific repair activity or is specifically recruited to damaged sites during mtDNA repair. This result is confirmed by more recent research showing that mtDNA breaks induce mtDNA replication in *Arabidopsis* (Qian et al. 2022)

### Gene expression reprogramming upon salt stress is altered in WHIRLY2 loss-of-function mutants

**Fig. 7:**
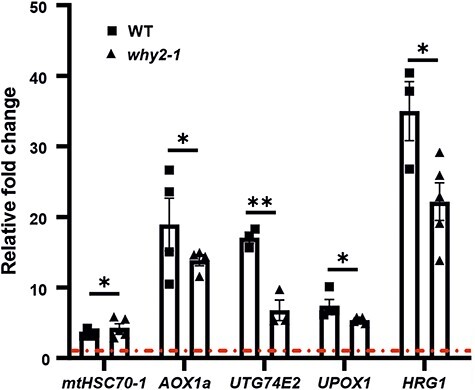
WHIRLY2 is necessary for proper mitochondria-to-nucleus communication. Relative gene expression of mitochondrial retrograde–regulated genes in WT and *why2-1* seedlings (14 DAS) treated for 8 h with 150 mM NaCl, compared to control conditions without NaCl (dash dot line). *ACT2* has been used as housekeeping gene. The results were analyzed using the 2^(−ΔΔCt)^ method. Error bars: SE. Statistical significance (*P*-value from one-way ANOVA followed by the Tukey test) is indicated by asterisks (*P < 0.05; **P < 0.005).

The morphology alterations of mitochondria are associated with general stress occurring at the level of mitochondria as indicated by the increased expression of the mitochondrial chaperon *mtHSC70-1* (**Fig. 7**). To acclimate to stress, plants must reprogram their gene expression and cellular metabolism to divert energy from growth and developmental processes toward stress responses. It is known that alteration in DNA metabolism of both chloroplasts and mitochondria can affect gene expression and genetic reprogramming in the nucleus (Cupp and Nielsen 2014). The organelles’ activity is closely coordinated with transcriptional activity in the nucleus through retrograde regulation, a feedback mechanism modifying nuclear gene expression to maintain or restore organellar functions. This intricate interplay ensures efficient cellular function and overall homeostasis. To investigate whether the lack of WHIRLY2 could compromise the signaling of stress from mitochondria to the nucleus, the expression profile of the marker genes under retrograde regulation has been investigated. One of the primary targets of retrograde regulation is the alternative oxidase (AltOx) which contributes to a reduction of ROS produced under conditions of mitochondrial dysfunction (Umbach et al. 2005). *UGT74E2, UPOX1* and *HRG1* are also identified as marker genes for mitochondrial dysfunction under retrograde control (Khan et al. 2023, Kacprzak et al. 2020). The gene expression data revealed that all the considered genes were induced upon salt stress in WT and the *why2-1* mutant. However, in the mutant line, their induction was significantly lower than that in WT (**Fig. 7**), indicating an apparent impairment in mitochondria–nucleus communication.

### Salt-induced Ca^2+^ dynamics

To assess whether WHIRLY2 disruption could impact stress perception, WT and *why2* mutant were compared in order to evaluate the calcium signature, which represents one of the earliest signaling pathways triggered by salt stress (Othman et al. 2017). According to Yang and Guo (2018), a rapid calcium uptake induced by salt activates the CBL/CIPK complex, which triggers sodium extrusion through specific transporters. A *why2-1* transgenic line harboring the Cameleon YC3.6 calcium probe (Behera et al. 2013) was generated, and in vivo cytosolic Ca^2+^ dynamics was analyzed in 5-day-old seedlings and in three different regions of the root (ROI 1, ROI 2 and ROI 3). Both WT and *why2* roots showed rapid Ca^2+^ transients upon salt addition and removal (**Supplementary Fig. S3**). The Förster resonance energy transfer (FRET) efficiency ratio calculated upon NaCl addition (IN) and removal (OUT) did not significantly differ between WT and mutant. These results suggest that salt sensitivity variance between WT and mutant is not linked to initial calcium signaling impairment triggered by salt perception.

### WHIRLY2 loss of function compromised the redox-dependent response triggered by salt stress

**Fig. 8:**
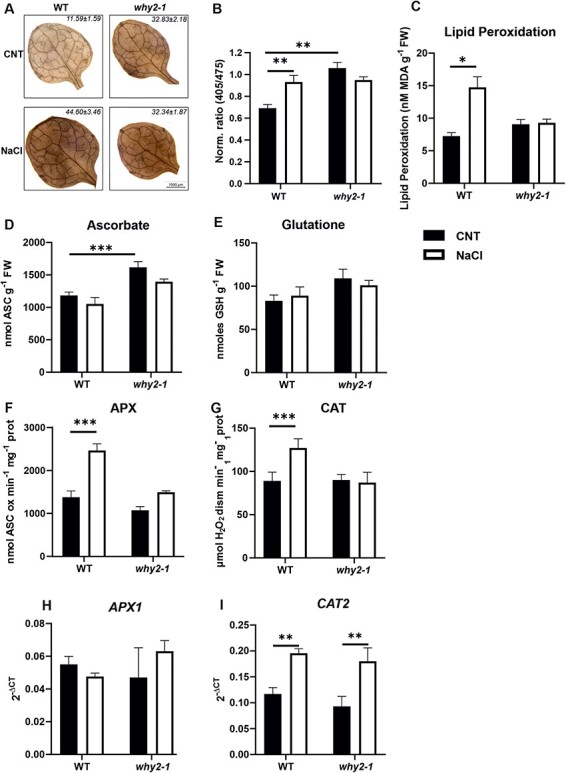
WHIRLY2 is necessary for proper redox-dependent response under salt stress. (A) Representative image of H_2_O_2_ accumulation, visualized by diaminobenzidine (DAB staining) in WT and *why2-1* mutant seedlings grown for 14 d and treated for 8 h with or without 150 mM NaCl in liquid medium. The analyses were repeated three times showing reproducible results. The percentage area (±SE) of 60 leaves (20 for each experiment) stained with DAB is reported inside the images. (B) H_2_O_2_ level detected in root in roGFP2-Orp1 sensor line WT and *why2-1* in response to 150 mM NaCl. Normalized average sensor ratios are plotted ± SE value (*n* = 9). (C) Lipid peroxidation, measured as MDA content, (D) total content of ASC and (E) total GSH, (F) total APX and (G) CAT activities in WT and *why2-1* mutant seedlings grown for 14 d and treated for 8 h with 150 mM NaCl. The values are plotted as ±SE (*n* = 5–7). (H and I) Expression profile of *Ascorbate peroxidases 1* and *Catalase2* on 14 DAS seedlings treated for 8 h with 150 mM NaCl. *ACT2* has been used as housekeeping gene. The results were analyzed using the 2^(−ΔCt)^ method. Statistical significance (*P*-value from the *t*-test) is indicated by asterisks (*P < 0.05; **P < 0.005; ***P < 0.001). Error bars: SE.

It is known that salt stress, through the activation of NADPH oxidases, can trigger ROS accumulation, activating a signaling cascade and downstream responses (Rejeb et al. 2015, Yang and Guo 2018). Thus, H_2_O_2_ accumulation was analyzed by dehydroascorbate (DAB) staining in the leaves of WT and *why2-1* mutant, grown under standard conditions or treated for 8 h with 150 mM NaCl. Under standard conditions, H_2_O_2_ accumulation was higher in the mutant line than in the WT (**Fig. 8A**). However, upon salt stress exposure, the level of H_2_O_2_ increased in WT, while it did not show substantial differences in the mutant line. H_2_O_2_ accumulation in the roots, measured by roGFP2-Orp1, showed the same behavior observed in the leaves, being higher in the *why2-1* mutant than in WT under control conditions and increasing only in WT in response to salt stress (**Fig. 8B**). Accordingly, lipid peroxidation, evaluated in the whole seedlings, did not vary significantly in response to salt stress in the *why2-1* mutant, whereas it increased in the WT (**Fig. 8C**). To characterize the redox-dependent defense response triggered by salt, the total content of the hydrophilic antioxidants, ascorbate (ASC) and glutathione (GSH), as well as the behavior of the two major enzymes involved in H_2_O_2_ scavenging, namely, catalase (CAT) and ASC peroxidase (APX), were analyzed in the whole seedlings (**Fig. 8**, **Supplementary Fig. S4**). Under control conditions, the ASC content was significantly higher in the *why2-1* mutant than in WT (**Fig. 8D**, **Supplementary Fig. S4A**); however, the total content of this antioxidant did not change after salt stress in both genotypes, whereby it remained higher in the mutant than in WT plants treated with NaCl. Total GSH content did not show any significant difference among the genotypes and in response to salt stress (**Fig. 8E**, **Supplementary Fig. S4B**). Total APX and CAT activities increased in the WT line after NaCl treatment. At the same time, they did not change significantly in the knock-out line (**Fig. 8F, G**, **Supplementary Fig. S4C, D**), showing a failure in activating ROS detoxification. To investigate whether the lack in the activity increase of H_2_O_2_ scavenging enzymes was due to an impairment of signaling, the transcript levels of the two genes *CAT2* and *APX1* were analyzed (**Fig. 8G, H**). After salt stress, *CAT2* transcript levels rose in both WT and mutant lines, whereas cytosolic *APX1* expression remained unaltered in both genotypes. (**Fig. 8H, I**). These results indicate that the lack of response to stress in terms of ROS scavenging system activation in mutant for *WHIRLY2* is not due to an alteration of the transcriptional regulation of detoxification enzymes.

## Discussion

The results of this study indicate that WHIRLY2 plays a crucial role in maintaining mtDNA integrity under stress conditions and that its disruption increases salt stress sensitivity in *Arabidopsis*.

Under standard conditions, the dramatic alterations at the mitochondrial level caused by the absence of WHIRLY2 do not have a noticeable impact on plant growth (Golin et al. 2020). However, under salt stress, mutants show more severe impairment in the germination rate and primary root growth compared to WT. Stress conditions, besides inducing widespread damage to cellular structures, including membranes and proteins, strongly impact the nucleoid structure and mtDNA integrity, which, in turn, activated mtDNA repair primarily involving WHIRLY2. Gene expression analyses in WT plants revealed indeed that expression of *WHIRLY2*, but not of other genes related to DNA repair in mitochondria, was significantly enhanced under salt stress conditions. Maintaining mtDNA integrity becomes imperative during stress response as it preserves mitochondrial functionality and, consequently, ensures efficient energy production. This agrees with the presence of stress-related regulatory sequences in the promoter of WHIRLY2. Under stress, the loss-of-function mutant did not repair damage in mtDNA, properly leading to the accumulation of aberrant recombination products that were not removed even after the stress subsided. The accumulation of aberrant mtDNA recombination fragments and the increased mtDNA synthesis were associated with a severe disturbance of nucleoid architecture and a profound alteration of the overall mitochondrial morphology. In the absence of WHIRLY2, the nucleoid architecture losing compactness may disrupt protein recruitment into complexes, causing improper replication and non-HR events. The data of this study revealed that WHIRLY2 is required to maintain mtDNA integrity because of its role in mtDNA repair (Marechal and Brisson 2010). In addition, the mtDNA integrity is necessary for nucleoid stability and, consequently, the maintenance of mitochondrial morphology and dynamics.

Mitochondria, besides being the primary powerhouse of the plant cell, play a role as environmental sensors being central to perceiving stress and orchestrating the cellular responses that allow the plant to cope with the stress (Liberatore et al. 2016). The absence of WHIRLY2 has a consequence not only on the alteration of mtDNA and nucleoid integrity but also on the efficiency of the communication between mitochondria and nucleus, as evidenced by the reduced stress-triggered transcriptional activation of nuclear genes that are under retrograde control. This interpretation follows the finding that the mutant plants are not impaired in stress perception and calcium-mediated signaling in the cytosol, while the redox-dependent response to stress was altered. First, it should be considered that under standard conditions, *why2-1* mutant shows a higher H_2_O_2_ level than WT. As previously shown in Golin et al. (2020), the lack of WHIRLY2 is associated with the disruption of mitochondrial internal structures with *why2-1* mutants showing nucleoid and cristae disorganization (Golin et al. 2020). The alterations can lead to disorganization of the mitochondrial electron transport (mtETC), resulting in altered electron flow and loss of *cytochrome c*, leading to an increase in ROS accumulation. Moreover, *why2-1* mutant compared to WT showed a higher ASC content. It is known that the last ASC biosynthetic step, catalyzed by l-galactono-1,4-lactone dehydrogenase (GALDH), is associated with the mtETC. The reaction provides an electron to *cytochrome c* (Bartoli et al. 2000), contributing to an over-reduction of the QH_2_ pool and hence an increased level of mitochondrial ROS, which indeed was observed in the mutant, under standard conditions. It is important to note that GALDH is considered a moonlighting protein that also plays a role in the structural regulation of the mtETC being a key assembly factor of complex I (Schimmeyer et al. 2016). WHIRLY2 is known to be associated with complex I too (Senkler et al. 2017), so it can be proposed that the absence of WHIRLY2 impairs the interaction between complex I and GALDH, impacting the architecture/stability of mtETC. This, in turn, might shift the role of moonlighting GALDH to its function in ASC synthesis (Phua et al. 2021). Thus, the high ASC level under standard conditions appears to be sufficient to prevent oxidative damage and preserve plant vitality. After salt exposure, H_2_O_2_ increase in WT plants leads to an enhancement of APX and CAT activities. In *why2-1* mutant, the high ASC content effectively prevents ROS accumulation and damage over a short period of time, as it is in plant exposed to salt for 8 h. However, in the absence of H_2_O_2_ accumulation, the mutant line failed to induce CAT and APX activities. Thus, the consequent lower ROS scavenging activity can impair the tolerance of plants to prolonged salt stress and could explain, at least in part, the elevated level of damage to the mtDNA under oxidative stress conditions. It is known that salt stress–derived injuries can be mitigated by a proper antioxidant response that is triggered by a reaction cascade that begins with an early H_2_O_2_ generation by NADPH oxidase. Indeed, mutants lacking the NADPH oxidase are more sensitive to salt stress (Rejeb et al. 2015). However, after salt stress, no difference was observed among the two genotypes in the transcriptional profile of *CAT2* and *APX1*. For these reasons, WHIRLY2 is likely involved in the regulation of the two enzymes responsible for H_2_O_2_ scavenging at the post-transcriptional level, thus providing protection from long salt stress–derived damages.

All these data taken together show that disruption of WHIRLY2 in the mutant line causes a pleiotropic phenotype, which might be primarily due to a compromised nucleus–mitochondria communication. Subsequently, the salt stress response, which includes the activation of those pathways that help the cell to buffer the damage and recover from the stress, is impaired. Our studies underline the importance of understanding the role of mitochondrial nucleoid architecture and integrity in stress response. Also, in humans/mammals, alterations of the mitochondrial nucleoid structure affect mitochondria function and mitochondrial–nuclear crosstalk, which is necessary to prevent severe diseases (Sharma et al. 2021). In plants, similar as in humans, mitochondrial nucleoids likely serve as a recruitment platform not only for oxidative phosphorylation complexes (Kim et al. 2021) but also for other proteins involved in the maintenance of mitochondria integrity and the regulation of stress signaling pathways.

## Materials and Methods

### Plant materials

The experiments were performed on *A. thaliana* plants, ecotype Columbia-0. Two different *whirly2* mutants of *A. thaliana* (ecotype Columbia-0) were used. WT and the *why2-1* mutant line SALKseq_118900.0 were obtained from the Nottingham Arabidopsis Stock Centre (NASC) and had been used in previous investigations (Maréchal et al. 2008, Golin et al. 2020). PCR analysis was performed to screen the plant collection and to check the insertion integrity. The *why2-3* mutant was obtained by CRISPR/Cas9–mediated genome editing (Li et al. 2021). Annealed oligos were integrated via *BbsI* restriction enzyme sites in plasmid pSI57 yielding pGH511. The gRNA/Cas9 expression cassettes were introduced into p6i-d35S-TE9 (DNA-Cloning-Service, Hamburg, Germany) via *SfiI*, generating plasmid pGH483. This construct was used for the *Agrobacterium*-mediated transformation of ecotype Col-0. Seeds were germinated on a hygromycin B–containing medium and screened for the presence of the T-DNA by specific primers as described (Li et al. 2021). Mutations in *WHIRLY2* were confirmed by Sanger sequencing. The *pWHY2::GUS* line was kindly provided by Qiang Cai from Peking University, China. *Arabidopsis thaliana* Col-0 lines harboring the Yellow Cameleon 3.6 NES biosensor cytosolic line (Loro et al. 2012) were crossed with the *why2-1* line to produce *why2* mutant lines harboring the different biosensors.

### Growth conditions

For in vitro grown plants, seeds were surface sterilized with 70% (v/v) ethanol supplemented with 0.05% (v/v) Triton-X 100 and 100% ethanol, plated on 1/2 Murashige Skoog (1/2MS) medium including vitamins (Duchefa-Biochemie B.V., Haarlem,The Netherlands) (Murashige and Skoog 1962), supplemented with 1% (w/v) sucrose and 0.5 g/l MES-OH, pH 5.8, and solidified with either 0.8% (w/v) plant agar or 1.5% (w/v) Phyto agar. After 48 h of stratification at 4°C, the plants were transferred in climatic chambers and grown under a long-day regime (16/8 h, 100 µmol photons m^−2^ s^−1^, 22°C and 65% humidity).

Treatments were performed on plants grown in 1/2MS medium either supplemented with 100 or 150 mM NaCl or with ciprofloxacin (0.25 and 0.75 µM) or treated for indicated times with the chemicals under different conditions.

### β-GUS analysis

Histochemical staining to detect GUS activity in plants harboring the construct *pWHIRLY2::GUS* (Cai et al. 2015) was performed by immersion of 12-day-old plants, treated for 24 h with 150 mM NaCl, in GUS staining solution [2 mM X-Gluc; 0.05% of Triton-X-100; 0.5 mM of K_3_(Fe(CN)_6_) × 3H_2_O; 0.5 mM of K_4_(Fe(CN)_6_) × 3H_2_O; 10 mM EDTA; 50 mM buffer phosphate, pH 7] overnight (O/N), at 37°C in the dark. Root samples were incubated at 37°C O/N and the day after washing in sterile distilled water. After the incubation period and the washing, tissue samples were collected and placed on microscope slides with 70 μl of imaging buffer (10 mM MES Tris base; 1 mM CaCl_2_; 5 mM KCl pH 5.8). GUS-stained plants were imaged using a stereomicroscope DMI 4000 and Leica DM6 B.

### Phenotype analyses

For root length analysis, photographs of vertically grown seedlings were taken at indicated time using a Bio-Rad ChemiDoc Touch Imaging System (Flamingo setting) and each image was processed using Fiji—ImageJ bundle software. The experiments were performed at least three times with three biological replicates, and each replicate involved 20 seedlings. For germination analysis, sterile seeds were sown on solid medium and grown horizontally. The images were acquired using a stereomicroscope (Leica MZ16F). Each image was processed using Fiji—ImageJ bundle software. The experiments were performed at least three times with three biological replicates, and each replicate included 36 seeds.

### Detection of DNA rearrangement products

Total DNA was extracted using the High-Quality DNA extraction kit following the protocol (Qiagen, Hilden, Germany). The samples were then analyzed by PCR using the *GoTaq* Polymerase (Merck KGaA, Darmstadt, Germany). For each reaction, 100 ng/μl of total DNA was used. DNA rearrangement events were detected using both outward- and inward-facing PCR primers spaced by ∼5–30 kb as previously described (Cappadocia et al. 2010). The sequence of the primer used in the analysis is reported in **Supplementary Table S1**. DNA was separated on 1.5% (w/v) agarose gel supplemented with GelRed. *Cox1* (ATMG01360) was used as a reference gene to normalize the DNA samples.

### mtDNA copy number assay

Total DNA was isolated with a High-Quality DNA extraction kit. To measure the amount of mtDNA, quantitative PCR (qPCR) was performed by using a mitochondrial gene (*Orf170mito;* AtMG00820) as a target and the nuclear‐encoded *RPOTP* gene (GenBank accession number Y08463) as an internal standard (Preuten et al. 2010) for nuclear genes. The mtDNA amount was calculated using the ΔCt method (Livak and Schmittgen 2001).

### qRT-PCR

One hundred milligrams of plant material was collected and ground in liquid nitrogen with mortar and pestle. Total RNA was extracted using the RNeasy® Plant Mini Kit (Qiagen) with an extra-protocol passage of 15 min of RNase-Free DNase (Qiagen) and resuspended in 30 μl of sterile nuclease-free water. RNA concentration was measured using a Nanodrop ND‐1000 spectrophotometer (Nano Drop Technologies Llc, Wilmington, Delaware, USA). First-strand cDNA synthesis was performed with a SuperScript-IV Reverse Transcriptase kit (Thermo Fisher Scientific, Waltham, Massachusetts, USA), using 2 μg of RNA and 1 μl of random primers (Sigma). qRT-PCR was performed in plate using Taq® qPCR Master Mix (Promega) with SYBR Green technology in either QuantStudio 12K Flex or QuantStudio 5 (Thermo Fisher Scientific, Waltham, Massachusetts, USA) instrument. The total volume of each reaction was 10 μl using 0.25 μl of primer mix (10 μmol). Sequences of the used primers are reported in **Supplementary Table S1**. *Arabidopsis* Actin-2 (*ACT2*; At3g18780) was used as internal control. The relative expression was calculated using the ΔCt and ΔΔCt method (Livak and Schmittgen 2001).

### Confocal imaging

For mitochondrial nucleoid analyses, seedlings were incubated for 4 min with 200 nM tetramethylrhodamine methyl-ester (TMRM) in imaging buffer (10 mM MES Tris base; 10 mM CaCl_2_; 5 mM KCl, pH 5.8) and washed for 10 min in imaging buffer. Stained samples were analyzed under the Confocal Laser Scanning Microscope Zeiss LSM700. For TMRM detection, samples were excited at 535 nm, and fluorescence was collected at 600 nm.

### Analyses of Ca^2+^ dynamics

*Arabidopsis* seedlings were grown vertically in 1/2MS solid medium for 7 d. Samples were then gently mounted on a perfusion chamber and stabilized with cotton wool soaked in 200 μl of imaging buffer (10 mM MES Tris base; 1 mM CaCl_2_; 5 mM KCl pH 5.8); an ISMATEC pump was used to administrate stimuli in continuous, setting the flow rate at 3 ml/min; each stimulus was added into the imaging buffer. The seedlings were kept under continuous perfusion. For Cameleon (Krebs and Schumacher 2013) analysis, the FRET CyanoFP/YellowFP (CFP/YFP) optical block A11400-03 (Emission 1, 483/32 nm for CFP and Emission 2 542/27 nm for the FRET) with a dichroic 510 nm mirror (Hamamatsu Photonics, Shizuoka, Giappone) was used for the simultaneous CFP and FRET acquisitions (cpVenus for YC3.6 and YC4.6). Images were acquired in 5-s intervals with an exposure time of 500 ms. Filters and dichroic mirrors were purchased from Chroma Technology, Olching, Germany. The NIS-Element (Nikon) was used to control the microscope, illuminator, camera, and post-acquisition analyses, as previously reported (Giovanna Loro et al. 2016). FRET efficiency values were calculated as previously described (Loro et al. 2013).

### Analyses of H_2_O_2_ root content

For H_2_O_2_ detection in the roots, we exploit the genetically encoded roGFP2-Orp1-probe (Nietzel et al. 2019). *Arabidopsis* seedling harboring the probe was crossed with *why2-1* mutant lines to generate mutant lines harboring the probe itself. *Arabidopsis* seedlings grown vertically, as previously described for 14 d, were incubated for 8 h in liquid medium [1/2MS medium including vitamins (Duchefa), with 1% (w/v) sucrose, 0.5 g/l MES-OH, pH 5.8] supplemented with 0 or 150 mM NaCl. Samples were then gently mounted on a chamber and stabilized with cotton wool soaked in 200 μl of the same incubation liquid medium, and the signal was acquired for 3 min. RoGFP2-Orp1 was excited sequentially at 405 and 488 nm, and emission was recorded at 505–535 nm. Images were acquired at 5-s intervals with an exposure time of 300 ms. The NIS-Element (Nikon) was used to control the microscope, illuminator, camera and post-acquisition analyses.

### TEM

TEM analyses were performed on plants collected at 23 d after sowing (DAS), which were grown in vitro on 1/2MS solid medium supplemented with 0 or 100 mM NaCl. Entire plants were fixed by incubating them O/N at 4°C in 2.5% (v/v) glutaraldehyde plus 2% paraformaldehyde (v/v) in 0.1M sodium cacodylate buffer, pH 7.4. The samples were post-fixed with 1% (w/v) osmium tetroxide for 2 h at 4°C. After three washes in water, the samples were dehydrated in ethanol and embedded in Epon resin (Sigma). Ultrafine sections (60–80 nm) of roots were obtained with a Leica Ultracut EM UC7 ultramicrotome, subsequently contrasted with 1% (w/v) uranyl acetate and 1% (w/v) lead citrate and visualized with a Tecnai G2 (FEI) transmission electron microscope operating at 100 kV. Images were captured with Velta (Olympus Soft Imaging System, Tokyo, Japan) digital camera.

### Determination of hydrogen peroxide and lipid peroxidation

In situ H_2_O_2_ accumulation in leaves was detected with DAB as described in Fortunato et al. (2022). ‘ImageJ’ software digitally acquired and quantified the staining intensity (https://imagej.nih.gov/ij/). The relative H_2_O_2_ levels were calculated as the percentage of DAB-stained area of leaves. The level of lipid peroxidation was evaluated in terms of malondialdehyde (MDA) content determined by thiobarbituric acid (TBA) reaction, as described by Paradiso et al. (2008). The amount of MDA-TBA complex was calculated using an extinction coefficient of 155 mM^−1^ cm^−1^.

### Analysis of enzymatic and non-enzymatic antioxidants

For ASC and GSH analyses, 0.3 g of samples was homogenized at 4°C with 1.8 ml of 5% (v/v) trichloroacetic acid. After centrifugation at 18,000×*g* for 20 min, the supernatants were collected and ASC and GSH levels were determined through the colorimetric assay described in de Pinto et al. (1999). For the determination of the activities of antioxidants enzymes, seedlings were ground in liquid nitrogen and homogenized at 4°C in a 1:8 (w/v) ratio with the extraction buffer (50 mM Tris–HCl pH 7.5, 0.05% (w/v) cysteine, 0.1% (w/v) bovine serum albumin, 1 mM phenylmethanesulfonyl fluoride). To determine APX activity, 1 mM ASC was added to the extraction buffer. Homogenates were centrifuged at 20,000×*g* for 15 min, and the supernatants were used for spectrophotometric and electrophoretic analyses. The protein concentration was determined according to Bradford (1976), using bovine serum albumin as a standard. CAT (EC 1.11.1.6) activity was spectrophotometrically measured according to Paradiso et al. (2020), and APX (APX, EC 1.11.1.11) activity was determined according to de Pinto et al. (1999).

### cis-Regulatory elements analysis

A sequence of 464 bp composed of the region upstream (344 bp), the first exon and the first intron (120 bp) of the gene *WHIRLY2*, was selected. The sequence was analyzed for *cis*-element prediction by using the Plant cis-acting regulatory DNA elements (PLACE) database (Higo et al. 1999).

### Statistical analysis

The values are represented as the means ± standard error. Asterisks describe the level of significance: *P < 0.05, **P < 0.005, ***P < 0.001 and ****P < 0.0001. Statistical significance was demonstrated using GraphPad Prism, performing the following tests: Student’s *t*‐test method, one-way ANOVA, Šídák’s multiple comparisons test and Tukey’s multiple comparisons test.

## Supplementary Material

pcae025_Supp

## Data Availability

The data underlying this article will be shared on reasonable request to the corresponding author.
