# Supplementary material for: The Arabidopsis Mitochondrial Nucleoid–Associated Protein WHIRLY2 Is Required for a Proper Response to Salt Stress
Source: Plant Cell Physiol. 2024 Mar 8;65(4):576–89. doi: 10.1093/pcp/pcae025 (PMC11094760; doi:10.1093/pcp/pcae025)
Supplement: pcae025_Supp [file pcae025_supp.zip › suppl_data/pcp-2024-e-00048-File010.pdf]

Supplementary. Information

Table S1. Lists of oligonucleotides used for the different analyses indicated.

Figure S1. In *why2-3* mutant mitochondrial morphology and seed germination are negatively affected. A primary root growth and B seed germination percentage of *why2-1* and *why2-3* compared to WT. C representative images of mitochondrial morphology analyses performed by confocal microscopy on root tissue cells of 5-day-old seedlings stained with 200nM TMRM. Arrows indicate elongated mitochondria morphology in mutant seedlings. Bar 20μm. D inflorescence length and E rosette area of *why2-3* compared to WT. F Electrophoretic analysis of representative PCR performed with 2 outward-facing mitochondria genome-directed PCR primers on total seedling DNA of WT, *why2-1* and *why2-3* plants grown for 23 days in solid medium supplemented with ciprofloxacin at different concentration. *Cox1* mitochondrial gene was used as loading control. The oligonucleotides used for each PCR are indicated. DAS: Days After Sowing. Statistical significance (p-value from t-test) is indicated by asterisks (**** = $p < 0.0001$). Error bars: SE.

Figure S2. Analysis of aberrant recombination product accumulation upon salt stress recovery. Electrophoretic analysis of representative PCR performed with 2 inward-facing mitochondria genome-directed primers. The *Cox1* mitochondrial gene was used as loading controls. The oligonucleotides used for each PCR are indicated. Plants were grown for 23 DAS in normal solid medium added with 150 mM NaCl and recovered for 10 days on standard solid medium. The experiment was repeated 3 times, 36 plants per biological replicate were used.

Figure S3. Cytosolic calcium signature is not impaired in *why2-1* mutant. A Representative image of $\Delta R/R0$ maximum variation over time in root tip of WT (left) and *why2-1* (right); black line indicates the treatment with NaCl 150 mM; FRET efficiency was determined over regions of interest (ROIs) which correspond to different root regions from tip (ROI1) to elongation zone (ROI3). B Statistical analyses of the value of $\Delta R/R0$ maximum variation during NaCl-addition (IN) or removal (OUT). Data were pooled for a statistical analysis with a one-way ANOVA followed by the Tukey test ($p<0.05$). N = 5; error bars = SD.

Figure S4. Salt stress in *why2-3* mutant does not induce a proper redox-dependent response. A total content of ascorbate (ASC) and B total glutathione (GSH), C total ascorbate peroxidase (APX) and D catalase (CAT) activities in WT and *why2-3* mutant seedlings grown for 14 days and treated for 8h ± with 150 mM NaCl in liquid medium. The values are the means ± standard errors of five independent experiments. Different letters indicate significant differences obtained by one-way ANOVA test ($p<0.05$).

Table S1. Lists of oligonucleotides

Accession number	Name	Analysis	Orientation	Sequence (5' - 3')
AT1G14410	*WHY1*	qRT-PCR analysis	For	ACTTCGAGAAGCAGAGGTTCGG
			Rev	TCTAGCAGGCAATCCTTCAGCAG
AT1G71260	*WHY2*	qRT-PCR analysis	For	GCATCCTCAAAACCAATGAC
			Rev	CATGATGTGTGGAAGAGCAA
AT2G02740	*WHY3*	qRT-PCR analysis	For	ACGATAGAACCACGAGCACCAG
			Rev	TGTCAGCTTGAACGCACCAGATTC
AT2G23320	*WRKY15*	qRT-PCR analysis	For	TCGTTGTCATTGCTCGAAGA
			Rev	CTTATCGCCGGAACCCTAAT
AT5G52310	*RD29A*	qRT-PCR analysis	For	TGCACCGGCTCATTCTGTAA
			Rev	GCAGAGAGACCGGAGTGTTC
AT1G71310	*ODB1*	qRT-PCR analysis	For	TCTTTGCCTTCTTGCCTCAGA
			Rev	ATTCCTTGACGGGTTTCATCAT
AT1G47720	*OSB1*	qRT-PCR analysis	For	ACGATTGGTGGGACAACAGGAGAA
			Rev	TCTGAGCAAAGCCAGAGAGCTTCA
AT5G44785	*OSB3*	qRT-PCR analysis	For	GGTCCTTGCTCCGATGGAAA
			Rev	GTCACATTTGGAGGAGGCGA
AT1G31010	*OSB4*	qRT-PCR analysis	For	GTACCAACCCAAAATCGCGA
			Rev	CCCCTTCAAACAGTACCGGA
AT3G24320	*MSH1*	qRT-PCR analysis	For	AGCATTATTTTCCCATGCTTGT
			Rev	TTTGCGCCCTCATCTAAACT
AT4G11060	*mtSSB1*	qRT-PCR analysis	For	ATCAAACCTCAACGACGTCG
			Rev	GCTCCTACAAGCCTCTGAT
AT3G18580	*mtSSB2*	qRT-PCR analysis	For	CCCCAGAAGACTCCGACTTT
			Rev	TTCCTGATCCCACCAGTTCC
AT3G20540	*Pol1B*	qRT-PCR analysis	For	CCTGAATACCGTTCACGTGCCCA
			Rev	AGCCGCACTTCCCTGAACAGGA
AT4G37910	*mtHSC70-1*	qRT-PCR analysis	For	GTCCAAATGGCTTCCGTATCTG
			Rev	CCAATAACATCATTCCCCACAG
AT3G22370	*AOX1a*	qRT-PCR analysis	For	GCCTACCGATTTGTTCTTCCAG
			Rev	CAGTGTAGTAACATTCCTCCAACCA
AT1G05680	*UGT74E2*	qRT-PCR analysis	For	ACAACAAAAACTAGAGTCAGTAACAAC
			Rev	TAACTTCTTCCACACTTCTCATAATCT
AT3G50930	*OM66*	qRT-PCR analysis	For	TGCTGAGACCAGGACGTATG
			Rev	ACCTTCCTCGATCTTGCTGA
AT2G21640	*UPOX1*	qRT-PCR analysis	For	AGAGGGTGAAGGATGATAACG
			Rev	GCTCCCGAATATCTTGTCCA
AT2G41730	*HRG1*	qRT-PCR analysis	For	CCACCTCCAACCGGCTCG
			Rev	ACTTGATAGCTGGCGACACG
AT1G71260	*WHY2*	Genotyping *why2-1*	For	CATGATGTGTGGAAGAGCAA
			Rev	GCATCCTCAAAACCAATGAC
	T-DNA		For	ATTTTGCCGATTTCGGAAC
AT1G71260	*WHY2*	Genotyping *why2-3*	For	CCTCAGAAGTCGGAAGACC
			Rev	TGCGAACAATCGACCACTAG
CAS9	*CAS9*	Genotyping *why2-3*	For	CAGCTCGTGCAGACCTACAAC
			Rev	TGCCTTCTAAGGATAGCGTG
mtDNA	*30001REV0*	mtDNA fragmentation	Rev	ACAGTCCACCAATAGCGGAAG
	80161FOR0		For	ACGTGCAAGTTTCCCTGCATG
mtDNA	*171214REV0*	mtDNA fragmentation	Rev	CATTCTAGCCCGAGAGGAACT
	180454FOR0		For	ACCTACCAGCCCCATGTAAAC
ATMG01360	*COX1*	mtDNA fragmentation	For	GCTAGCTCATGGCAGGAAATC
			Rev	GTAACGTCCGTTCCGTGATCT
ATMG00820	*orf170 mito*	mtDNA copy number	For	CTTTAGCAACCAAGCGAGCC
			Rev	TGATGCTCTCTCTCGGAACA
AT2G24120	*AtRpoTp*	mtDNA copy number	For	TGGAAGCCGTCTGCTAGAACTA
			Rev	TGTCTGAATGCAGGTCGAAAC

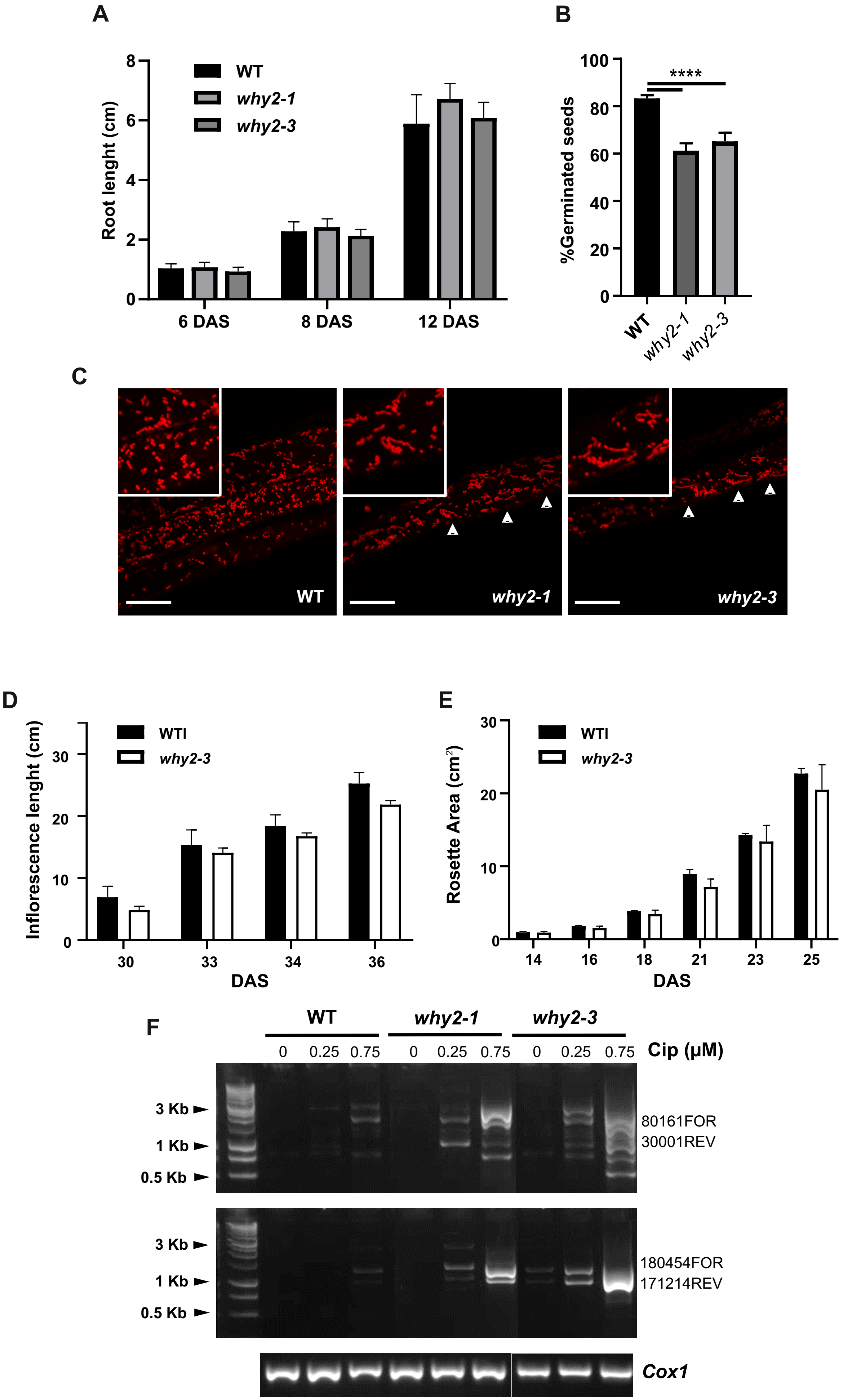
A
Root lenght (cm)
WT
why2-1
why2-3
6 DAS
8 DAS
12 DAS
B
%Germinated seeds
****
WT
why2-1
why2-3
C
WT
why2-1
why2-3
D
Inflorescence lenght (cm)
WTl
why2-3
DAS
E
Rosette Area (cm2)
WTl
why2-3
DAS
F
WT
why2-1
why2-3
Cip (μM)
3 Kb
1 Kb
0.5 Kb
80161FOR
30001REV
180454FOR
171214REV
Cox1

figure S1

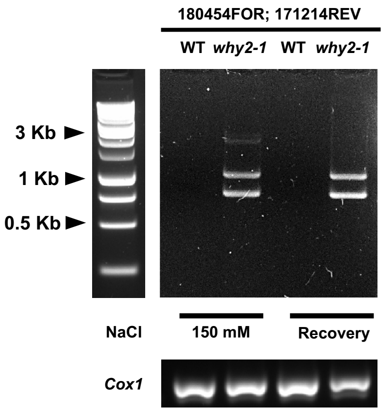
180454FOR; 171214REV
WT
why2-1
WT
why2-1
3 Kb
1 Kb
0.5 Kb
NaCl
150 mM
Recovery
Cox1

figure S2

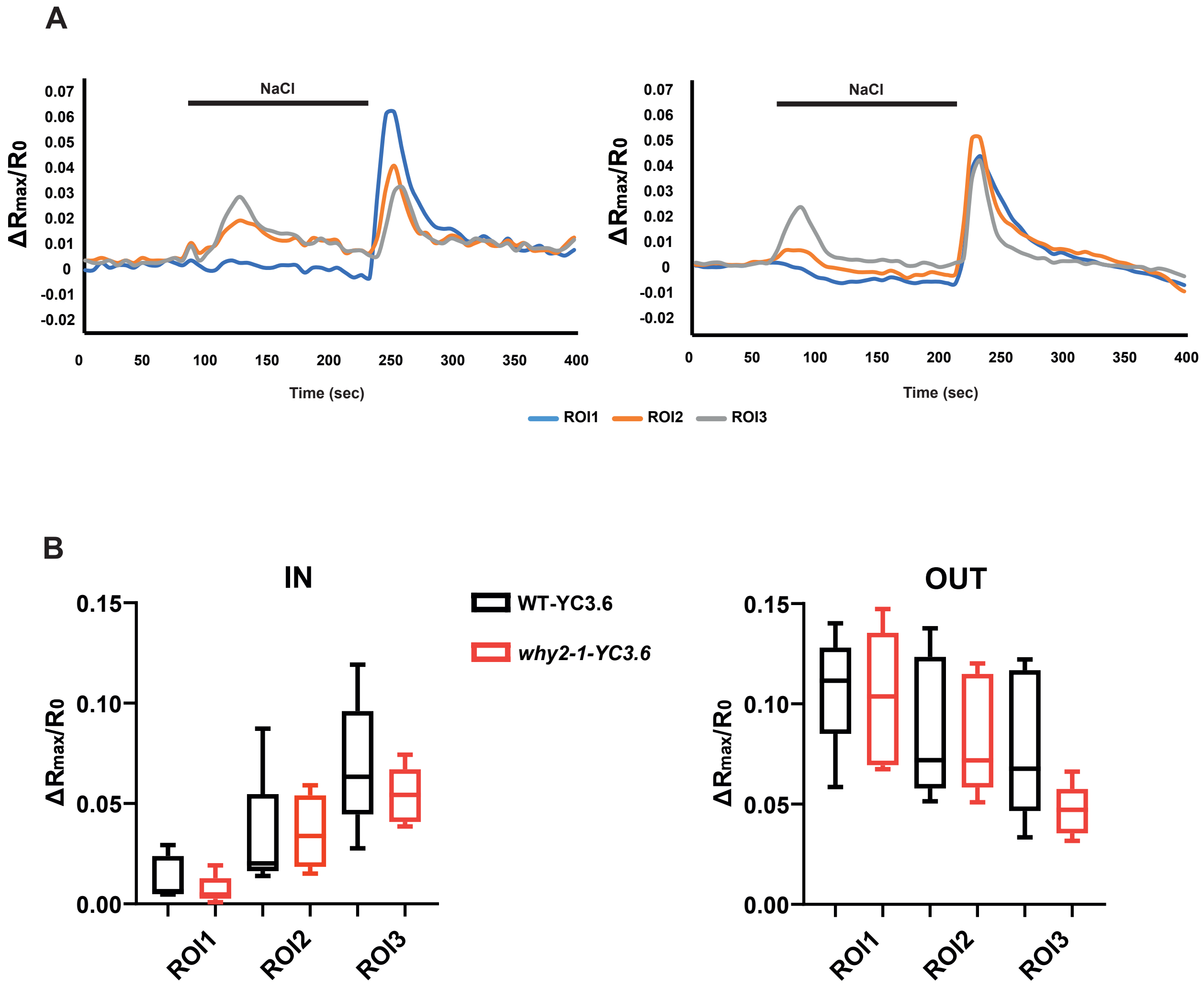
A
NaCl
ΔRmax/R0
0.07
0.06
0.05
0.04
0.03
0.02
0.01
0
-0.01
-0.02
0
50
100
150
200
250
300
350
400
Time (sec)
ROI1
ROI2
ROI3
B
IN
OUT
WT-YC3.6
why2-1-YC3.6
0.15
0.10
0.05
0.00
ROI1
ROI2
ROI3

figure S3

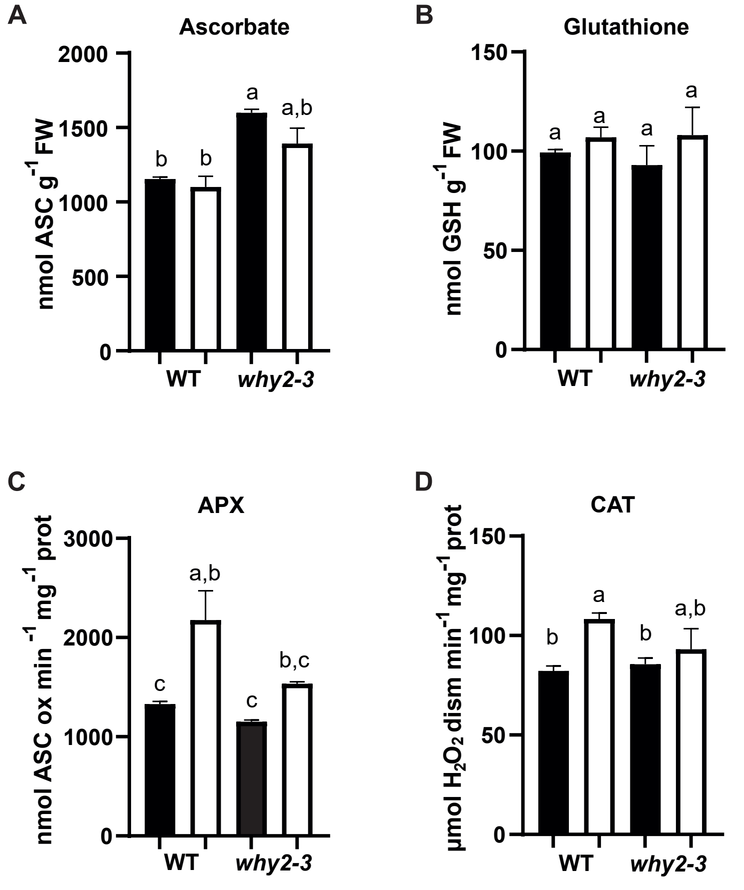

A
Ascorbate
nmol ASC g-1 FW
2000
1500
1000
500
0
b
b
a
a,b
WT
why2-3
B
Glutathione
nmol GSH g-1 FW
150
100
50
0
a
a
a
a
WT
why2-3
C
APX
nmol ASC ox min-1 mg-1 prot
3000
2000
1000
0
c
a,b
c
b,c
WT
why2-3
D
CAT
µmol H2O2 dism min-1 mg-1 prot
150
100
50
0
b
a
b
a,b
WT
why2-3

figure S4
